# Predictive models for acute kidney injury in acute pancreatitis: a systematic review and meta-analysis

**DOI:** 10.3389/fmed.2025.1699717

**Published:** 2026-02-05

**Authors:** Haoran Zhu, Feifei Li, Shengteng Guo, Yijun Xiao, Wentao Zhu, Qinghua Wang

**Affiliations:** 1 School of Nursing, Binzhou Medical University, Binzhou, Shandong Province, China; 2 Department of Critical Care Medicine, Binzhou Medical University Hospital, Binzhou, Shandong, China; 3 Department of Hepatobiliary Surgery, Binzhou Medical University Hospital, Binzhou, Shandong, China

**Keywords:** acute pancreatitis, acute kidney injury, predictive modeling, systematic review, meta-analysis

## Abstract

**Background:**

The utilization of predictive models facilitates the identification of patients at risk, thereby enabling the implementation of individualized interventions. Despite the growing use of predictive models to estimate the likelihood of AKI in AP, concerns persist regarding their effectiveness in clinical settings and the rigor and relevance of forthcoming research. The objective of this study is to systematically review and evaluate predictive models for AKI in AP.

**Methods:**

A comprehensive search of relevant databases was conducted, encompassing China National Knowledge Infrastructure (CNKI), Wanfang, VIP, Chinese Medical Association, PubMed, Web of Science, Scopus, and Cochrane Library, with the search extending from database inception to 26 November 2024. The data from a number of selected studies was extracted using the CHARMS form, while the quality of predictive modeling studies was assessed using the PROBAST tool. A meta-analysis of AUC for predictive models and relevant predictors (≥2) was conducted using Stata 17.0 and MedCalc software.

**Results:**

The total number of studies included in the review was 17, with a total of 9,949 patients and 37 predictive models. Of these, 32 models underwent internal validation, with an area under the curve (AUC) > 0.7. The overall risk of bias was high across all 17 studies, yet the overall applicability was deemed satisfactory. The results of the meta-analysis indicated that the pooled AUC for internal validation across 20 predictive models for AKI in AP was 0.790 (95% CI = 0.761–0.818); and the pooled external validation AUC for five models was 0.766 (95% CI = 0.684–0.845). The overall risk of bias was high across all 17 studies, with significant heterogeneity observed. However, the overall applicability was deemed satisfactory.

**Conclusion:**

The predictive model for AKI complicating AP demonstrates moderate predictive efficacy. Nevertheless, given the elevated risk of bias in the majority of studies and the absence of adequate external validation, its clinical applicability merits further investigation.

**Systematic review registration:**

https://www.crd.york.ac.uk/PROSPERO/view/CRD420251008769, identifier CRD420251008769.

## 1 Introduction

Acute pancreatitis (AP) is a common inflammatory disease of the gastrointestinal tract and a leading cause of hospitalization for digestive disorders. It is characterized by sudden abdominal pain and elevated pancreatic enzymes. The revised Atlanta classification categorizes AP into mild (MAP), moderately severe (MSAP), and severe (SAP) forms based on clinical severity (1). The global incidence of AP is rising by 2 to 5% annually, reaching 34 cases per 100,000 people each year (2). Around 20% of patients develop SAP, which is marked by systemic inflammatory response syndrome (SIRS) and multiple organ failure, resulting in high morbidity and mortality (3). The pathogenesis of AP involves premature activation of pancreatic enzymes, leading to tissue autodigestion and local or systemic complications (4). Among these, acute kidney injury (AKI) is both common and life-threatening. It is driven by multifactorial mechanisms, including hypovolemia, cytokine storms (e.g., IL-6, TNF-*α*), SIRS, and reduced renal perfusion due to pancreatic necrosis. AKI occurs in 10–42% of AP patients, with mortality rates up to 80% (5, 6). Although several scoring systems and biomarkers have been proposed to predict AP-related AKI, their performance is limited by confounders and reliance on static clinical parameters. These tools often lack specificity for kidney injury and fail to reflect disease progression dynamically, resulting in unsatisfactory predictive accuracy (7, 8).

In this context, prediction models integrating multiple variables have gained increasing attention. They offer a more individualized and risk-based approach to care (9). In 2009, Xue et al. (10) used the least absolute shrinkage and selection operator (LASSO) to identify 13 key radiomics features, building a model to predict AP progression and improve early outcomes. Subsequent studies have focused on AP-AKI models. Yang et al. (11), for instance, used logistic regression to show that C-reactive protein, intra-abdominal pressure, and cystatin C were strongly associated with AKI risk. With advances in big data and artificial intelligence, machine learning has shown promise in diagnosis (12), complication monitoring (13), and prognosis (14). Artificial neural networks (ANNs) can identify patterns from clinical or imaging data to support decision-making and improve diagnostic accuracy (15, 16). Many AP-AKI prediction models have since been proposed to enable early risk identification and guide timely intervention. However, their performance varies, and model quality is often evaluated inconsistently using metrics like calibration curves, decision curve analysis (DCA), clinical impact curves, and the Hosmer–Lemeshow test. The area under the curve (AUC) remains the most widely used metric (17), but no meta-analysis has yet synthesized AUC values across studies. Moreover, considerable heterogeneity in predictors affects model generalizability and clinical utility, which requires systematic investigation.

Although numerous prediction models have been developed for AP complicated by AKI, no systematic review or meta-analysis has evaluated their characteristics or clinical utility. This study aims to synthesize and compare existing models in terms of key features, predictors, modeling methods, and performance. It also assesses methodological quality to support the development or selection of reliable tools for early AKI risk assessment in AP.

## 2 Methods and analysis

### 2.1 Defining the research question

Guided by the PICOTS framework, following systematic review guidelines for prediction models, the research question was structured around the following evidence-based components: the target population (P) was defined as adult patients diagnosed with acute pancreatitis; the index prediction model (I) of interest was designed to assess the risk of developing AKI following AP; no comparator model (C) was specified; the primary outcome (O) consisted of the occurrence of AKI; the prediction window (T) was limited to within 2 weeks after AP onset; and the intended setting (S) for model application was the intensive care unit.

### 2.2 Search strategy

This systematic evaluation was conducted in accordance with the Preferred Reporting Items for Systematic Reviews and Meta-Analyses (PRISMA) guidelines (18). A comprehensive literature search was conducted in the China National Knowledge Infrastructure (CNKI), Wanfang, Wipro (VIP), Chinese Medical Association (CMA), PubMed, Web of Science, Scopus, and Cochrane Library databases. The search period for these databases extended from their inception to 8 November 2025.

To minimize publication bias, a dedicated search for gray literature was conducted using a predefined strategy. For Chinese gray literature, we searched the CNKI Doctoral and Master’s Dissertation Database, China Conference Paper Full-text Database, China National Medical Research Registry, and National Science and Technology Achievements Network. For English gray literature, the search included OpenGray, ProQuest Dissertations & Theses Global, WHO Global Index Medicus, ClinicalTrials.gov, EU Clinical Trials Register, and NIH Internal Research Reports. The same Chinese and English keywords used for database searches (e.g., “acute pancreatitis,” “acute kidney injury,” “predictive model”) were applied to gray literature retrieval. The search period for gray literature also ranged from database inception to November 8, 2025. Additionally, we attempted to obtain unpublished data by contacting 3 leading experts in acute pancreatitis and 5 research teams with relevant published studies, requesting access to ongoing or completed but unpublished research on predictive models for AKI in AP.

Despite these efforts, no gray literature was included in the final analysis for two reasons. First, most retrieved gray literature (e.g., dissertations, conference abstracts) lacked key information required for our analysis, such as detailed methods for predictive model construction, validation processes, or outcome data (e.g., sensitivity, specificity, area under the curve). Second, the contacted experts and research teams could not provide usable data due to ongoing research, data confidentiality agreements, and unresolved intellectual property issues.

The search strategy included a broad range of keywords to ensure the inclusion of all studies on predictive models for AKI in AP. Chinese keywords were: pancreatitis, acute pancreatitis, acute kidney injury, predictive model. English keywords were: acute pancreatitis, acute kidney injury, acute kidney failure, acute kidney insufficiency, predictive model, predictive score, predictive risk, predictive value. In addition, the references of included studies were manually searched to identify additional relevant research. Detailed search strategies, including those for gray literature, are available in the Supplementary materials.

### 2.3 Study selection

Inclusion criteria: (1) Patients with AP combined with AKI were included in the study. ① Diagnostic criteria for acute pancreatitis: According to the Atlanta International Consensus Criteria (1), patients with AP were classified into three types: MAP, MSAP and SAP; ② Diagnostic criteria for acute kidney injury: the Risk, Injury, Failure, Loss, End-stage Kidney Disease (RIFLE) criteria, the AKI Network (AKIN) criteria or the Kidney Disease Institute for Global Prognosis Improvement (KDIGO) criteria (19). The RIFLE criteria classify AKI into 5 stages: risk, injury, failure, loss and end-stage renal disease (ESRD); the AKIN criteria classify AKI into 3 stages: risk, injury and failure; the KDIGO criteria classify AKI into 1 stage based on creatinine and urine volume. Urine volume to classify AKI into stages 1, 2 and 3 (20). (2) The study was about the construction and empirical evidence of a prediction model for outcomes related to AP combined with AKI; (3) The study type was observational study (cohort study, case–control study).

Exclusion criteria: (1) duplicate publications, reviews, animal experiments, conference abstracts, duplicate published studies; (2) literature with inaccessible full text, incomplete data, incorrect data or inaccessible data. (3) Non-Chinese and non-English literature.

### 2.4 Data extraction

The retrieved literature was imported into EndNoteX9, and two researchers independently performed literature screening by first reviewing the titles and abstracts. Those studies that met the inclusion criteria were included in the next round of full-text evaluation. A methodological review of the full text of the articles was conducted. Information was extracted from eligible studies and then cross-checked. Disagreements were resolved by a second evaluator through a consensus process. The researcher developed a form based on the Critical Appraisal and Data Extraction for Systematic Reviews of Prediction Modeling Studies (CHARMS) (21) to conduct literature data extraction. The data extraction form encompassed the following: the first author, the year of publication, the specimen size, the modeling method, the area under the subject’s operating characteristic curve, the sensitivity and specificity, the method of model validation, and the final predictors included.

### 2.5 Assessment of methodological quality

The Risk of Bias Assessment Tool for Predictive Modeling of Risk of Bias and Clinical Applicability Studies (Prediction Model Risk of Bias Assessment Tool, PROBAST) (22) was utilized to evaluate the 17 included papers. Two investigators independently performed the risk of bias and applicability evaluation of the literature. In the event of disagreement, the matter was discussed and resolved in consultation with a third investigator. The matter was resolved through negotiation.

#### 2.5.1 Risk of bias assessment

The PROBAST risk assessment tool is comprised of four domains, including study population, predictors, outcomes and statistical analyses. It involves 20 signature questions to evaluate the risk of bias and applicability of predictive modeling studies. Each of the four domains of risk of bias assessment comprises a series of between two and nine signature questions, with the results of each domain being categorized as low, high, or unclear. In addition, the answers to each signal question are limited to “Yes,” “probably Yes,” “No information,” “No,” or “probably No.” Responses such as “No information,” “No,” or “probably No” are to be expected. A series of specific assessments were conducted to ascertain the risk of bias. In instances where all questions were answered with “Yes/probably Yes,” the risk of bias was deemed low. Conversely, if at least one question was answered with ‘No/probably No’, the risk of bias was considered high. If the response was “No information” for the given question, but “Yes/probably Yes” for the other questions, the risk of bias was deemed unclear.

#### 2.5.2 Assessment of applicability

The evaluation of suitability is conducted within three distinct domains: the study population, the predictors, and the outcomes. The methodology employed is analogous to that of risk of bias, however, there is an absence of a signature problem in each domain, which is categorized as “low risk of suitability,” “high risk of suitability,” or “unclear.” The term ‘unclear’ is employed to denote an absence of clarity or precision in the specified subject or text. The evaluation of the overall study was based on an assessment of applicability in the first three domains, and the study was categorized as low risk of suitability, high risk of suitability, or unclear.

### 2.6 Analysis

A meta-analysis of the AUC of the MSAP and SAP-complicated AKI prediction model and the associated predictors of patients (study ≥2) in the included studies was performed by applying Stata17.0 and MedCalc software. The effect statistics of the predictors were expressed as SMD and OR (95% CI), with *p* < 0.05 being considered as a significant difference. Data expressed in median form were converted to mean form using an online data conversion tool developed by the Department of Mathematics at Hong Kong Baptist University.^1^ The heterogeneity among studies was judged using the I^2^ test. When heterogeneity among studies was not significant (*p* > 0.05, I^2^ < 50%), a fixed-effects model was used. Conversely, sensitivity analyses were first performed by the one-by-one exclusion method. If heterogeneity was still high (*p* < 0.05, I^2^ ≥ 50%), a random-effects model was used for the merger.

## 3 Results

### 3.1 Study selection

**Figure 1:**
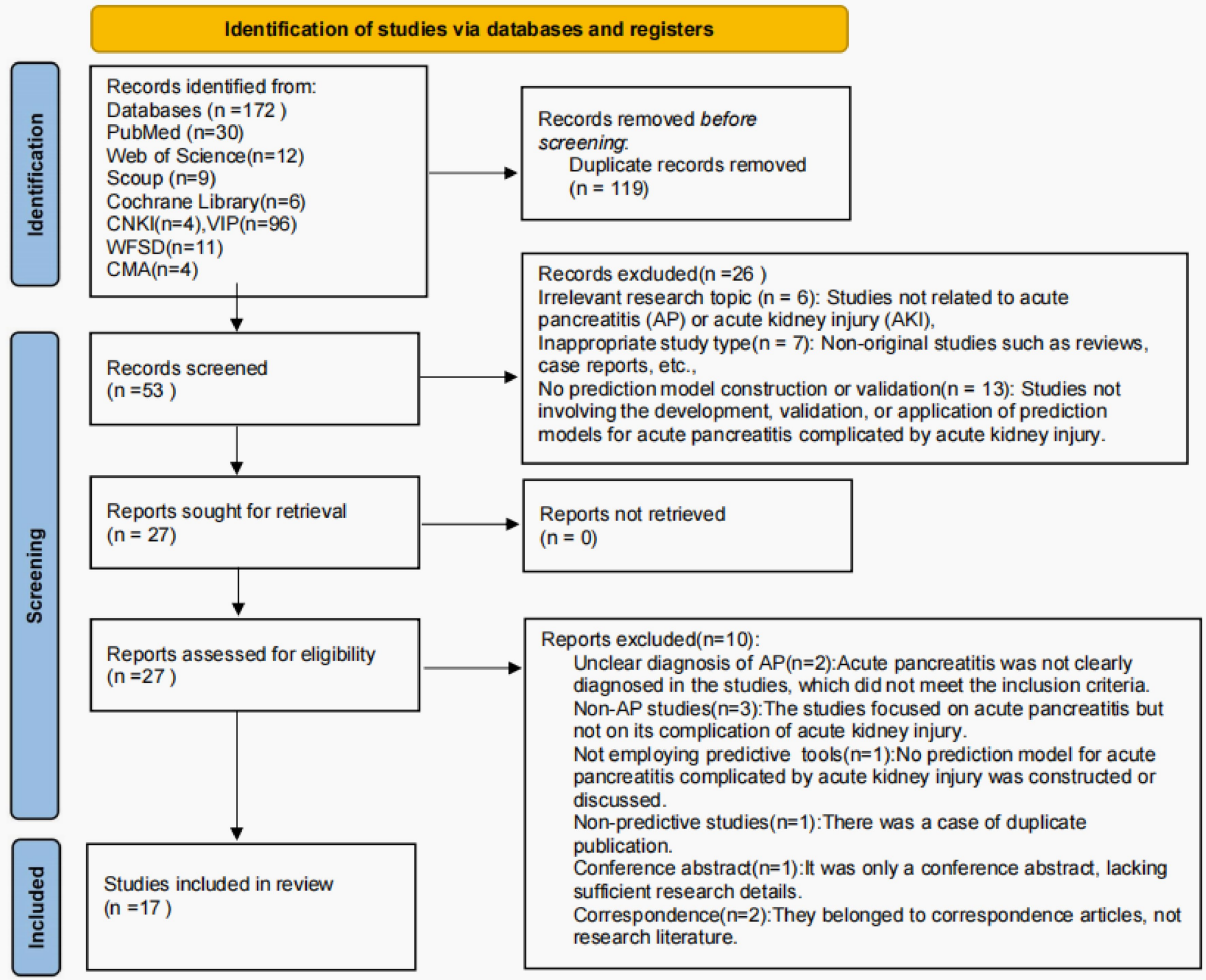
Literature screening flow chart. CNKI, China National Knowledge Infrastructure; VIP, Wipro; CMA, Chinese Medical Association.

A total of 172 documents were retrieved, including those from the following sources: PubMed (*n* = 30), Web of Science (*n* = 12), Scopus (*n* = 9), Cochrane Library (*n* = 6), CNKI (*n* = 4), VIP (*n* = 96), Wanfang (*n* = 11), and CMA (*n* = 4). In conclusion, a total of 17 articles were included in the review, encompassing 11 articles in English and 6 in Chinese. The process of literature screening and the subsequent results are illustrated in Figure 1.

### 3.2 Characteristics of the included studies

To summarize the characteristics of the included research literature, mostly published in 2019–2024. The sample size of each study ranged from 110 to 1,265. The number of models ranged from 1 to 7. A total of 9,949 patients and 37 predictive models were included in this systematic evaluation. There were a total of 17 studies were retrospective single-center studies; one study (23) was a retrospective two-center study; two studies (24, 25) were single-center prospective studies; and two studies (26, 27) were retrospective multicenter studies. 2 studies (11, 28) were prediction models for AKI complicated by MSAP and SAP, 2 studies (29, 30) were prediction models for AKI complicated by SAP, 1 study (31) was a risk assessment model for AKI complicated by AP, 1 study (32) was a prediction model for AKI complicated by acute hyperlipidemic pancreatitis, and the rest were prediction models for AKI complicated by AP. The characteristics of the included studies are shown in Table 1. Additionally, a summary table of key characteristics for the clinical usability of predictive models is provided in the Supplementary file.

**Table 1:** Basic characteristics of included literature.

Included literature	Year of publication	Country	Study design	Participants	Data source	Primary outcome	AKI (cases)	Sample size	AKI incidence (%)
Yang et al. (11)	2022	China	Retrospective study	≥ 18 years, AP patients	A tertiary general hospital	AKI	202	996	20.28
Qu et al. (38)	2020	China	Retrospective study	≥ 18 years, AP patients	A tertiary general hospital	AKI	80	334	23.95
Yun (33)	2019	China	Retrospective study	≥ 18 years, AP patients	A tertiary general hospital	AKI	83	308	26.95
Yue et al. (29)	2022	China	Retrospective study	≥ 18 years, AP patients	A tertiary general hospital	AKI	61	295	20.68
Yang et al. (23)	2022	China	Retrospective study	≥ 18 years, AP patients	2 tertiary general hospitals	AKI	67	424	15.80
Jiang et al. (31)	2023	Israel	Retrospective study	≥ 18 years, AP patients	MIMIC database	AKI	695	963	72. 17
Shang et al. (34)	2023	China	Retrospective study	≥ 18 years, AP patients	A tertiary general hospital	AKI	62	450	13.78
Chen et al. (28)	2023	China	Retrospective study	≥ 18 years, AP patients	A tertiary general hospital	AKI	140	437	32.04
Wu et al. (35)	2023	Israel	Retrospective study	≥ 18 years, AP patients	MIMIC database	AKI	499	799	62.45
Ying and Yu (36)	2024	China	Retrospective study	≥ 18 years, AP patients	A tertiary general hospital	AKI	64	249	25.70
Chi et al. (24)	2024	China	Retrospective study	≥ 18 years, AP patients	A tertiary general hospital	AKI	79	258	30.62
Zhang et al. (16)	2024	China	Prospective study	≥ 18 years, AP patients	4 tertiary medical centers	AKI	374	772	48.45
Chi et al. (24)	2024	China	Prospective study	≥ 18 years, AP patients	A tertiary general hospital	AKI	79	258	30.62
Lin et al. (25)	2024	America	Retrospective study	≥ 18 years, AP patients	MIMIC database	AKI	667	1,235	54.00
Liu et al. (30)	2024	China	Retrospective study	≥ 18 years, AP patients	MIMIC-IV database, eICU-CRD, Xiangya Hospital of Central South University	AKI	150	1,089	13.77
Yuan et al. (26)	2024	China	Retrospective study	≥ 18 years, AP patients	3 tertiary general hospitals	AKI	138	672	20.53
He et al. (27)	2024	China	Retrospective study	≥ 18 years, AP patients	A tertiary general hospital	AKI	23	110	20.90

### 3.3 Construction of predictive models

A total of 17 papers reported 37 models for predicting the risk of MSAP and SAP complicating acute kidney injury. With regard to the modeling methods employed, 11 studies (11, 25, 28, 29, 31–37) utilized Logistic Regression (LR) to construct predictive models, while 6 studies (23, 24, 26, 27, 30, 38) employed Machine Learning (ML) for this purpose. With regard to the selection of variables, 11 studies (11, 24, 28, 29, 31–37) were based on multifactor analysis. With regard to the administration of continuous variables, it was observed that all studies retained the continuity of these variables, thus maintaining the integrity of the continuous variable treatment. In the case of missing data, three studies (27, 30, 35) utilized multiple interpolation to impute missing values, three studies (11, 24, 31) excluded missing data, and the remaining 11 studies did not explicitly report the presence of missing data (Table 2).

**Table 2:** Characteristics of the 17 studies.

Author	Year	Sample size	Modeling methods		AUC (95% CI)		Sensitivity / Specificity		Validation method	Predictive factors	Calibration method	Missing data processing methods
			Optimal method	All methods	Training set	Test set (I/E)	Training set	Test set				
Yun (33)	2019	533	LR	LR	0.971		0.920/0.900	-	-	CRP, ALB, APACHE II, PO2, CA^2+^.	-	-
Qu et al. (38)	2020	334	XGBoost	LR	0.873		0.607/0.864		Ten-fold cross-validation	APACHE II, IAP, PCT.	-	-
				CART	0.803		0.619/0.833				Calibration plot, DCA	Delete
				XGBoost	0.919		0.619/0.882				DCA, CIC	-
				SVM	0.863		0.536/0.849				Calibration plot, Hosmer-Lemeshow	Delete
				RF	0.882		0.476/0.847				Calibration plot, DCA	-
Yue et al. (29)	2022	295	LR	LR	0.987	I:0.976	0.990/0.985	0.986/0.942	-	APACHE II, Ranson, Scr, PCT, CysC, CA^2+^	Calibration plot, DCA	-
Yang et al. (11)	2022	996	LR	LR	0.993	I:1.000			Cross-Validation	IAP, CRP, CysC	Calibration plot, DCA	Multiple imputation
Yang et al. (23)	2022	424	RFC	RFC	0.902(0.400–1.403)	I:0.913(0.364–1.462)	-	-	10-fold cross-validation	CRP, PLR, NAR, NLR, Scr, CysC.	Calibration plot, Hosmer-Lemeshow	-
				SVM	0.725(0.223–1.227)	I:0.758(0.209–1.307)	-	-			Hosmer-Lemeshow	-
				DT	0.887(0.385–1.389)	I:0.891(0.342–1.440)	-	-			Calibration plot	Delete
				ANN	0.874(0.370–1.374)	I:0.868(0.339–1.397)	-	-			-	-
				XGBoost	0.791(0.289–1.293)	I:0.801(0.272–1.330)	-	-			-	Multiple imputation
Jiang et al. (31)	2023	963	LR	LR	0.820(0.790–0.860)	I:0.760(0.700–0.820)	-	-	-	Weight, Sepsis, CHF, SOFA, Wbc, Alb	Calibration plot, Brier score,	Multiple imputation
Shang et al. (34)	2023	450	LR	LR	0.745(0.710–0.780)		-	-	Bootstrap	Male, SAP, Hypoproteinemia, Diabetes, Obesity	Calibration plot, Brier score	-
Chen et al. (28)	2023	565	LR	LR	0.944	I:1.000	-	-	-	CRP, IAP, CysC.	-	-
Wu et al. (35)	2023	799	LR	LR	0.795(0.758–0.832)	I:0.772 (0.711–0.832)			internal validation	Age, Ethnicity, T-BIL, APTT Vasoactive drugs, Sepsis.	Calibration method	Missing data Processing methods
Ying and Yu (36)	2024	355	LR	LR	-	I:0.892(0.853–931)/E:0.904(0.867–0.940)	-	-	internal validation	Scr, BUN, CRP, NLR, APACHE II.	-	-
Chi et al. (24)	2024	258	LR	LR	0.856(0.790–0.922)	-	0.798/0.967	-	-	Age, Tyg, PCT.	-	-
Zhang et al. (16)	2024	437	DL	GBM	-	I:0.801(0.705–0.812)	-	0.759/0.811	5-fold cross-validation	Cr, ALB, LHD.	-	-
				DRF	-	I:0.800(0.696–0.904)	-	0.853/0.806			Calibration plot, DCA	Delete
				GLM	-	I:0.734(0.630–0.838)	-	0.768/0.742			DCA, CIC	-
				DL	-	I:0.830(0.734–0.926)	-	0.832/0.815			Calibration plot, Hosmer-Lemeshow	Delete
				LR		I:0.799(0.694–0.905)	-	0.586/0.947			Calibration plot, DCA	-
Chi et al. (24)	2024	258	LR	LR	0.856 (0.790–0.922)	-	0.796/0.967	-	-	Age, TyG, PCT.	Calibration plot, DCA	-
Lin et al. (25)	2024	1,235	GBM	GBM	0.814 (0.763–0.865)	I:0.867 (0.831–0.903)	0.715/0.751	0.800/0.788	10-fold cross-validation	Urine volume, Mechanical ventilation, Wbc, Vasoactive drugs, Mean heart rate, Mean respiratory rate, Maximum creatinine levels.	Calibration plot, DCA	Multiple imputation
				GLM	0.812 (0.769–0.854)	I:0.849 (0.810–0.888)	0.720/0.736	0.825/0.741			Calibration plot, Hosmer-Lemeshow	-
				KNN	0.671 (0.622–0.719)	I:0.688 (0.634–0.741)	0.589/0.663	0.705/0.571			Hosmer-Lemeshow	-
				NB	0.812 (0.780–0.864)	I:0.831 (0.790–0.872)	0.871/0.429	0.850/0.671			Calibration plot	Delete
				NNET	0.688 (0.624–0.752)	I:0.753 (0.704–0.802)	0.701/0.611	0.775/0.624			-	-
				RF	0.809 (0.766–0.851)	I:0.859 (0.823–0.886)	0.7100/0.744	0.780/0.782			-	Multiple imputation
				SVM	0.810 (0.763–0.856)	I:0.856 (0.818–0.894)	0.688/0.780	0.805/0.771			Calibration plot, Brier score,	Multiple imputation
Liu et al. (30)	2024	1,265	XGBOOST	LR	0.788(0.767–0.808)	E:0.691(0.613–0.796)	0.646/0.802	0.406/0.825	10-fold cross-validation	Age, Neutrophils, RDW, BUN, AlB, SBP, RRT, Vasopressor.	Calibration plot, Brier score	-
				XGBoost	0.941(0.931–0.952)	E:0.724(0.648–0.800)	0.935/0.815	0.568/0.323	-		-	-
				RF	0.894(0.880–0.908)	E:0.677(0.599–0.756)	0.856/0.754	0.5966/0.4271	-	-	Calibration method	Missing data Processing methods
Yuan et al. (26)	2024	672	XGBOOST	XGBOOST	0.900(0.780–0.990) 0.860(0.800–0.960) 0.820(0.790–0.980)	E:0.81(0.73–0.89)	-	-	10-fold cross-validation	PA_WV_LHL_GLDM_GLV, PA_WV_LLL_NGTDM_Complexity, PA_WV_LHH_GLSZM_SZN, PA_WV_LHL_GLRLM_RV, EP_WV_LHH_GLSZM_SZN, EP_WV_LLH_GLRLM_LRE.	-	-
He et al. (27)	2024	110	LR	LR	0.875(0.972–1.000)	-	1.000/0.957		-	VExUS Score, TyG Index.	-	-

Cr, Creatinine; ALB, Albumin; LHD, lactatedehydrogenase; APACHE II, Acute physiology and chronic health evaluation II; SOFA, Sequential organ failure assessment; CRP, C-reactive protein; WBC, White blood cell count; PCT, Procalcitonin; CysC, Cystatin C; IAP, Intra-abdominal pressure; CHF, Chronic heart failure; NAR, Neopterin-to-tryptophan ratio; SCr, Serum Creatinine; RDW, Red Cell Distribution Width; SBP, Systolic Blood Pressure; RRT, Renal Replacement Therapy; PLR, Platelet-to-Lymphocyte Ratio; T-BIL, Total bilirubin; APTT, Activating Partial Thrombin Time; BUN, Blood Urea Nitrogen; NLR, Neutrophil-to-Lymphocyte Ratio; PA, Pancreatic region; EP, Peripancreatic region; GLDM, Gray Level Dependence Matrix; NGTDM, Neighboring Gray Tone Difference Matrix; GLCM, Gray Level Co-occurrence Matrix; GLV, Gray Level Variance; GLSZM, Gray Level Size Zone Matrix; SZN, Size-Zone Non-Uniformity; GLRLM, Gray Level Run Length Matrix; RV, Run Variance; ZV, Zone Variance; LRE, Long Run Emphasis; SZN, Size-Zone Non-Uniformity; DV, Difference Variance; DE, Difference Entropy; TyG, Triglyceride-glucose; VExUS, Venous excess ultrasound; XGBoost, Extreme gradient boosting; LR, Logistic regression; GBM, Gradient Boosting Machine; RF, Random Forest; DL, Deep Learning; CART, Classification And Regression Trees; SVM, Support Vector Machine; RFC, Random Forest Classifier; DT, Decision Tree; ANN, Artificial Neural Network; DRF, Distributed Random Forest; GLM, Generalized Linear Model; KNN, K-Nearest Neighbors; NB, Naive Bayes; NNET, Neural Network; AUR, Area under the receiver; I, Internal Validation; E, External Validation. -, Unclear.

### 3.4 Predicting model performance

The included models were evaluated for their ability to discriminate using AUC of the subjects’ work characteristics. However, it is important to note that five studies (24, 25, 35, 37, 38) failed to report AUC values at the time of modeling for the models. The remaining literature provides AUC at the time of modeling, which ranges from 0.745 to 0.987. Thirteen studies (11, 23–27, 30, 31, 33–35, 37, 38) report the internal validation AUC of the model, which ranges from 0.724 to 1.000. Two studies (33, 34) report the external validation AUC of the study, which ranges from 0.810 to 0.904, and the calibration of the model was evaluated using the Hosmer-Lemeshow test, calibration curves, and decision curve analyses. Eight studies (11, 24, 26–28, 31, 34–36) reported calibration; three studies (28, 36, 37) reported the Hosmer-Lemeshow test, and five studies (11, 23, 28, 34, 35) reported the decision curve analysis. In order to validate the model, 13 studies (11, 23–28, 30, 31, 35, 36) conducted internal validation of the model and 2 studies (26, 36) conducted internal and external validation of the model (Table 2).

### 3.5 Risk of bias and applicability assessment

The overall risk of bias for all 17 studies was categorized as high risk of bias. Subject domain: 15 studies (11, 26–38) were retrospective and thus exposed to a high risk of bias, while two studies (29, 33) were prospective and also exposed to a high risk of bias. In the domain of prediction, 21 studies were found to be at low risk. In the domain of clinical outcomes, a total of 21 studies were classified as low risk in the predictive domain. Analysis domain: 14 studies (11, 23–26, 28, 31–38) exhibited a high risk of bias due to inadequate handling of missing data, while four studies (32–34, 37) demonstrated a high risk of bias stemming from the absence of internal validation procedures (Table 3). The overall assessment indicated that all studies exhibited a high risk of bias, yet the predictive models demonstrated a commendable degree of adaptation.

**Table 3:** Quality assessment scores of the 17 studies.

Study	Year	Risk of bias				Applicability			Overall	
		Participants	Predictors	Outcome	Analysis	Participants	Predictors	Outcome	Risk of bias	Applicability
Yun (33)	2019	H	L	L	H	L	L	L	H	L
Qu et al. (38)	2020	H	L	L	H	H	H	L	H	L
Yue et al. (29)	2022	H	L	L	H	L	L	L	H	L
Yang et al. (11)	2022	H	L	L	H	H	H	L	H	L
Yang et al. (23)	2022	H	L	L	H	H	L	L	H	L
Jiang et al. (31)	2023	H	L	L	H	L	L	L	H	L
Shang et al. (34)	2023	H	L	L	H	L	L	L	H	L
Chen et al. (28)	2023	H	L	L	H	L	L	L	H	L
Wu et al. (35)	2023	H	L	L	L	H	H	L	H	L
Ying and Yu (36)	2023	H	L	L	H	L	L	L	H	L
Chi et al. (24)	2024	H	L	L	H	L	H	L	H	L
Zhang et al. (16)	2024	H	L	L	H	H	L	L	H	L
Chi et al. (24)	2024	H	L	L	H	H	H	L	H	L
Lin et al. (25)	2024	H	L	L	L	H	H	L	H	L
Liu et al. (24)	2024	H	L	L	L	L	L	L	H	L
Yuan et al. (26)	2024	H	L	L	H	L	L	L	H	L
He et al. (27)	2024	H	L	L	H	H	H	L	H	L

L, low risk of bias/low concern regarding applicability; H, high risk of bias/high concern regarding applicability.

### 3.6 Meta-regression

#### 3.6.1 AUC results

Due to the underreporting of model validation details in the included studies, a total of 20 models from 11 studies (11, 23, 24, 26–31, 34–36) were found to be suitable for merging of internally validated AUC; 5 models from 3 studies (27, 28, 36) were found to be eligible for merging of externally exploratory analysis AUC (Table 2).

To ensure the rigor of the analytical approach, this study employed three key strategies for the meta-analysis, namely logit (AUC) transformation, a variance-weighted random-effects model, and Hartung-Knapp adjustment. Specifically, logit (AUC) transformation effectively mitigates the boundary effects and non-normal distribution issues inherent to AUC values; the variance-weighted random-effects model is well-suited for synthesizing heterogeneous datasets; and the Hartung-Knapp adjustment corrects biases in effect size estimation under high heterogeneity scenarios. All these approaches align with the Cochrane Collaboration’s recommendations for meta-analyses of diagnostic tests.

**Figure 2:**
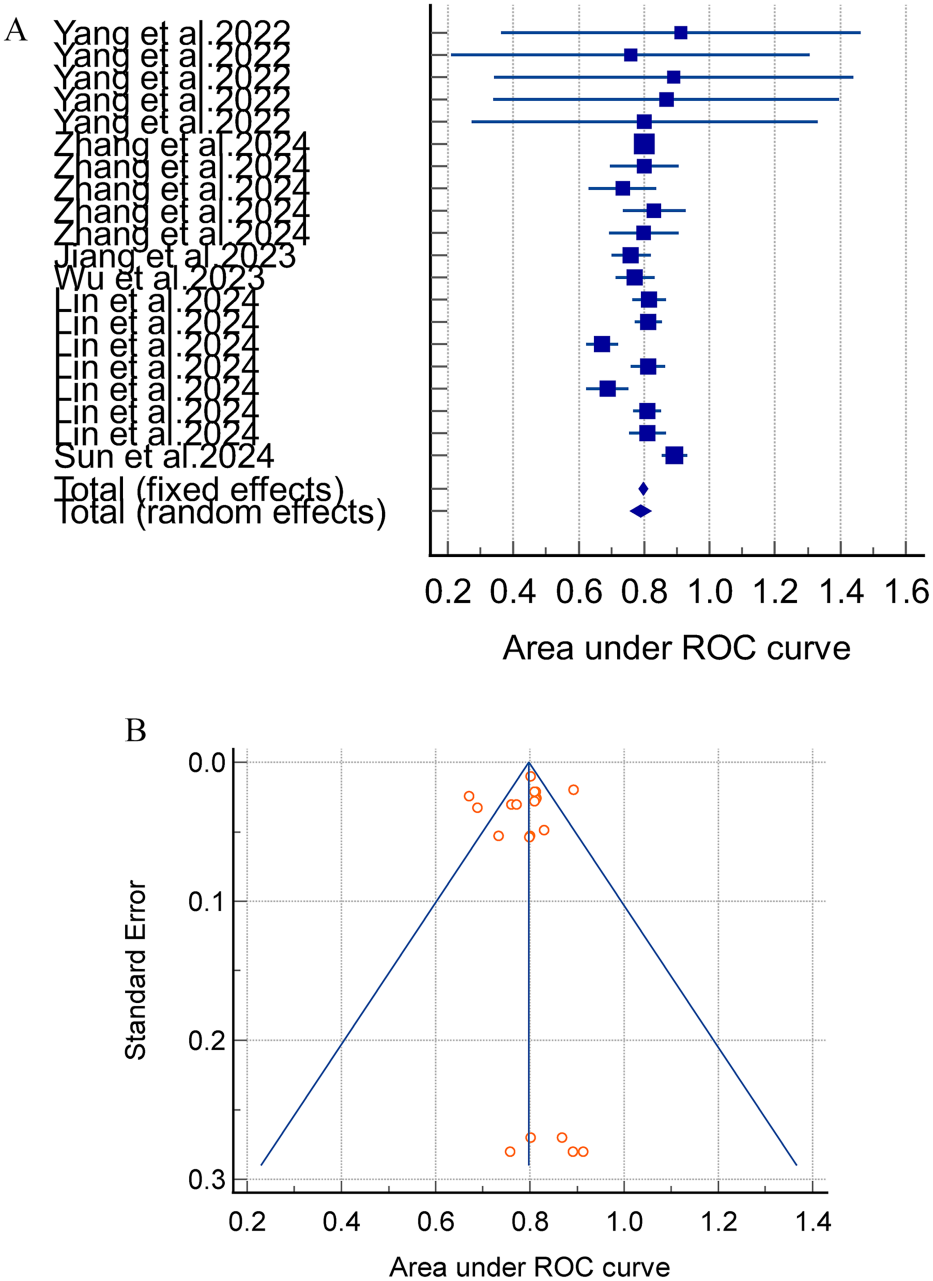
**(A)** The AUC of internal validation for the acute pancreatitis with acute kidney injury prediction model. **(B)** Avisual funnel plot for assessing potential publication bias in internal validation within a meta-analysis.

Meta-analyses using a random-effects model yielded an internally validated merged AUC of 0.812 (95%CI = 0.783–0.840, I^2^ = 75.40%, *p* < 0.0001) and an external exploratory analysis merged AUC of 0.766 (95% CI = 0.684–0.845, I^2^ = 92.03%, *p* < 0.0001). These findings suggest a substantial heterogeneity between studies. The combined internal and external validation AUC values demonstrated stability in the sensitivity analyses (Figure 2).

Notably, the separate analysis of internally and externally validated AUC values is methodologically valid in this study. However, the external validation subgroup comprised merely 3 studies with 5 models, and this small sample size directly led to extremely high heterogeneity (I^2^ > 90%). Consequently, the pooled results of external validation AUC should be categorized as exploratory rather than confirmatory findings, and their inferential power requires cautious interpretation.

**Figure 3:**
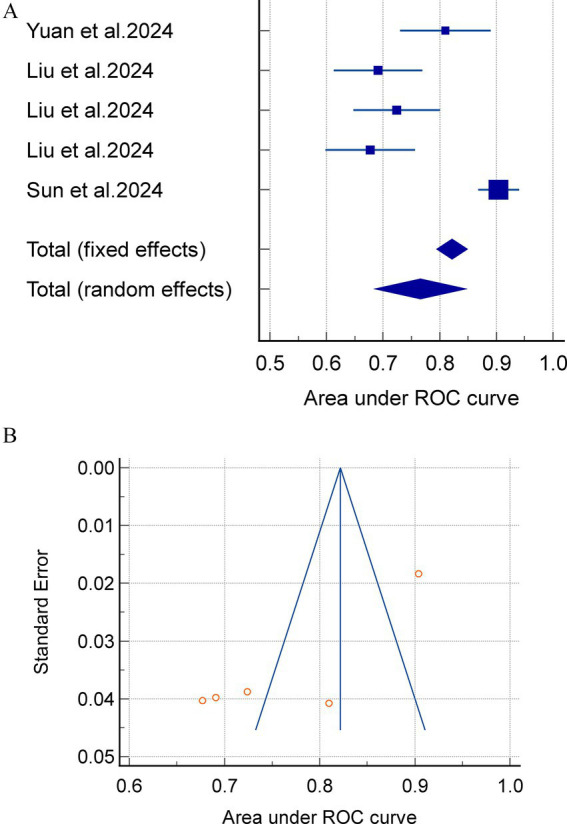
**(A)** A meta analysis of the external exploratory AUC for the acute pancreatitis with acute kidney injury prediction model. **(B)** Avisual funnel plot for assessing potential publication bias in external validation within a meta-analysis.

Subgroup analyses were conducted based on the various model types, with 20 models reporting internally validated AUC values. Four studies (24, 31, 35, 36) incorporating four linear models (LR and LASSO) reported a combined AUC of 0.811 (95% CI = 0.751–0.870, I^2^ = 84.08%, *p* = 0.0004), and three studies (23, 24, 30) involving 16 nonlinear models (RFC, SVM, XGBOOST, DL, etc.). The results of the meta-analysis revealed a combined AUC of 0.812 (95% CI = 0.779–0.844, I^2^ = 74.10%, *p* < 0.0001). Sensitivity analyses were performed on the models with high heterogeneity by means of one-by-one exclusion. The results of the sensitivity analyses for each subgroup showed instability, indicating that the meta-analysis results were not robust (Figure 3).

#### 3.6.2 Predictive factors

A meta-analysis was conducted, which revealed that Age, IAP, Ca^2+^, PCT, APACHE II, CRP, CysC, ALB, TYG, SCr, BUN and NLR were all predictors of the prediction model for AP complicating AKI (*p* < 0.05). The results of the meta-analysis are shown in Table 4.

**Table 4:** Meta-analysis of predictors for acute kidney injury complicating acute pancreatitis.

Predictive factors	No. studies included	No. patients	Heterogeneity		Effect model	Effect size	95%CI	*p*
			I^2^(%)	*p*				
Age	2	516	0.0	1.0000	Random	SMD = 0.84	(0.64–1.03)	<0.0001
IAP	3	1895	84.6	0.0020	Random	SMD = 2.31	(2.18–2.44)	<0.0001
Ca^2+^	2	603	0.0	0.8150	Random	SMD = -0.91	(−1.10–−0.71)	<0.0001
PCT	4	1,145	86.6	<0.0001	Random	SMD = 1.43	(1.30–1.96)	<0.0001
APACHE II	3	937	90.7	<0.0001	Random	SMD = 1.69	(1.52–1.86)	<0.0001
CRP	5	2,414	99.3	<0.0001	Random	SMD = 1.92	(1.80–2.04)	<0.0001
CysC	4	2,152	99.2	<0.0001	Random	SMD = 2.48	(2.34–2.61)	<0.0001
ALB	2	621	98.6	<0.0001	Random	SMD = -0.35	(−0.54–−0.16)	<0.0001
TyG	3	626	61.1	0.0760	Random	SMD = 0.36	(0.19–0.54)	<0.0001
SCr	3	840	98.7	<0.0001	Random	SMD = 1.49	(1.30–1.68)	<0.0001
BUN	2	1,484	93.6	<0.0001	Random	SMD = 0.71	(0.60–0.82)	<0.0001
NLR	2	545	0.0	0.3490	Random	SMD = 1.56	(1.33–1.79)	<0.0001

IAP, Intra-abdominal pressure; PCT, Procalcitonin; APACHE II, Acute physiology and chronic health evaluation II; CRP, C-reactive protein; CysC, Cystatin C; AlB, Albumin; TyG, Triglyceride-glucose; SCr, Serum Creatinine; BUN, Blood Urea Nitrogen; NLR, Neutrophil-to-Lymphocyte Ratio.

### 3.7 Sensitivity analysis

The findings of the sensitivity analysis demonstrated that the heterogeneity of IAP, PCT, and TYG index diminished following the exclusion of a particular literature source (28, 29, 38), respectively. The outcomes indicated that the results of the meta-analysis were unstable, suggesting that the influence of three literature sources was substantial. The heterogeneity of CRP, CysC, and SCr did not show any significant change after the exclusion of one literature source, which indicated that the results of the meta-analysis had a better stability and high reliability. The system has been demonstrated to exhibit high reliability. The number of literature sources incorporated into the study for ALB and BUN predictors was two, and the one-by-one exclusion method was not applicable for conducting sensitivity analysis. The specific results of sensitivity are demonstrated in the Supplementary material.

## 4 Discussion

A comprehensive review of the current literature identified 17 studies reporting 37 prediction models for AP complicated by AKI. Among them, 11 studies involving 32 models underwent internal validation. Of these, 20 models reported complete AUC values with 95% confidence intervals, yielding a pooled AUC of 0.790 (95% CI: 0.761–0.818). In contrast, five models from three studies underwent external exploratory analysis, with complete AUC reporting and a pooled AUC of 0.766 (95% CI: 0.684–0.845). These results indicate that the overall discriminatory ability of AP-AKI prediction models is moderate. Notably, models with internal validation demonstrated higher pooled AUCs than those with external exploratory analysis. This discrepancy can be explained by differences in validation methodology. Internal validation assesses model performance using the same dataset employed during model development, which may lead to overfitting and inflated performance estimates. In contrast, external exploratory analysis relies on independent datasets that may differ in population characteristics, data quality, and confounding factors, thereby providing a more rigorous assessment of model generalizability.

Beyond discrimination, calibration represents another fundamental metric for evaluating clinical prediction models, as it reflects the alignment between predicted probabilities and observed outcomes (39). Our systematic review indicates that while most models exhibited adequate discriminatory ability (AUC > 0.7), comprehensive assessment of calibration was largely insufficient. Few studies systematically applied well-established calibration methods, such as calibration plots, Hosmer-Lemeshow tests, or Brier scores. The absence of robust calibration data not only introduces potential bias but may also substantially limit a holistic understanding of overall model performance. Notably, significant methodological heterogeneity exists across included studies—particularly in terms of validation strategies and calibration assessment—which may pose substantial challenges to evidence synthesis and clinical translation.

A critical appraisal of the validation methods used in these studies further highlights potential methodological vulnerabilities. A prevalent practice is the reliance on split-sample validation, wherein the original dataset is simply divided into training and testing subsets. This approach, while straightforward, has inherent limitations that render it relatively superficial. A key concern is its inability to fully account for “optimism bias” inherent in model development: since the testing subset is derived from the same population as the training data, it may not fully simulate the variability encountered in real-world clinical settings, thereby tending to inflate performance estimates and mask potential overfitting risks. This limitation may be exacerbated by the fact that split-sample validation provides only a single snapshot of model performance, often lacking the statistical robustness needed to quantify the stability of model predictions. In contrast, more rigorous resampling methods—such as bootstrapping (which generates multiple synthetic datasets through repeated sampling with replacement) or k-fold cross-validation (which partitions data into k subsets and iteratively uses k-1 subsets for training and 1 for testing)—may effectively mitigate such optimism. By leveraging the full dataset for both model development and validation, these approaches can offer a more realistic reflection of a model’s performance.

This variability in validation rigor—with most studies opting for the convenient yet less robust split-sample approach over rigorous resampling techniques—can complicate cross-study comparisons and meta-analyses, potentially leading to overestimated expectations of model performance in external populations. Furthermore, deficiencies in validation rigor often coincide with inadequate calibration assessment: studies that adopt less rigorous validation methods also tend to neglect systematic calibration analysis. This trend results in inconsistent reporting of calibration metrics (e.g., most studies fail to provide calibration plots, slopes, or intercepts), which may undermine the objective assessment of predictive accuracy. Without establishing standardized evaluation frameworks that simultaneously encompass both discrimination and calibration, and encourage the use of robust validation methodologies, the translation of predictive models into clinical practice is likely to remain hindered. Therefore, future research should consider adopting unified reporting standards that mandate comprehensive performance metrics, while implementing internal-external validation schemes to support reliable evidence synthesis and meaningful clinical application.

In the present study, six investigations (23, 24, 26, 27, 30, 38) incorporated machine learning (ML) algorithms into the development of their predictive models. Notably, study (25) compared the predictive efficacy of these ML-based models against established scoring systems, namely the BISAP, Ranson, and APACHE II scores. The findings revealed that ML-driven predictive models markedly surpassed those leveraging logistic regression (LR) in conjunction with the Ranson, APACHE II, and SOFA scores, particularly in forecasting outcomes related to AP complicated by AKI. When juxtaposed with ML, LR exhibits certain limitations (40). As a prevalent linear classifier, LR is prone to being confounded by nonlinear interactions among predictors and tends to exhibit inferior accuracy relative to ML, especially when dealing with high-dimensional feature spaces. Yuan et al. (26) amalgamated machine learning algorithms with radiological features to construct a predictive model, thereby unlocking the substantial potential of machine learning in the screening of acute pancreatitis and facilitating personalized treatment and management strategies for AP patients. As the volume of recorded patient features continues to escalate, ML’s capacity to harness high-dimensional data enables it to unravel the intricate relationships among multiple features (41), thereby enhancing predictive precision. Moreover, extant research (42, 43) has underscored the variability in predictive performance of machine learning models across diverse patient cohorts and datasets. The criteria for selecting salient predictive indicators also diverge among similar studies. Consequently, the development of clinical prediction models must be meticulously aligned with the data characteristics and pragmatic requirements of specific medical contexts to optimize the equilibrium between accuracy and clinical applicability.

Meta-analysis of predictors identified across the included models revealed several commonly used variables, including age, IAP, Ca^2+^, PCT, APACHE II score, CRP, CysC, ALB, TYG, SCr, BUN, and NLR.

The included studies demonstrated considerable heterogeneity in sample size, ranging from 110 to 1,265 participants. Of the 17 studies, 11 (64.7%) had a sample size ≤500 cases, while only 3 exceeded 900 cases. Notably, small-scale studies (24, 31) consistently reported relatively high AUC values (0.856–0.875), with some approaching or exceeding 0.90—a pattern that contrasts with the effect sizes of large-scale studies (23, 25). For instance, Yang et al. (23) (*n* = 996) reported an AUC of 0.993, whereas Lin et al. (25) (*n* = 1,235) documented an AUC of 0.814 for the GBM model. This discrepancy reflects the potential “small study effect,” wherein studies with limited sample sizes and statistical power are more prone to positive result bias: accidental findings or selective reporting may inflate diagnostic performance indicators to meet publication criteria. A notable example is He et al.’s (27) 110-participant study, where the 95% CI for AUC was unusually narrow (0.972–1.000)—a result lacking clinical plausibility. This is likely a statistical artifact caused by insufficient sample representativeness, rather than a true reflection of diagnostic efficacy. These biases not only amplify overall heterogeneity (consistent with the previously reported high I^2^ values) but also introduce the risk of overestimating the pooled effect size in the meta-analysis, which aligns with sensitivity analysis findings that specific studies (31, 35) exerted a significant influence on the overall effect size.

Building on the “small study effect,” the current body of research on AP-AKI prediction models exhibits systemic methodological limitations throughout the model development workflow, collectively compromising the reliability of reported high AUC values. Most studies failed to meet the well-validated criterion of at least 10 events per predictor variable (EPV ≥ 10) (44), with small-sample studies (27) often integrating multiple predictors—violating the “predictor number × 10 × 4” principle and undermining model stability. Additionally, nine studies (23, 25, 28, 29, 32–34, 36, 37) did not report blinding for predictor/outcome assessment, and two prospective studies (24, 25) with high AUCs suffered from small sample sizes (*n* = 258 and 334) and inadequate missing data handling (45), increasing subjective and selection bias. Many studies (23–27, 30, 32–38) also relied on univariate variable selection, which ignores predictor interactions and risks model misspecification, while logistic regression failed to address multicollinearity—an issue resolvable via LASSO regression (46). Beyond these systemic flaws, a prominent manifestation lies in selective reporting of validation results: 14 studies used internal validation (e.g., cross-validation, Bootstrap resampling) and reported high AUCs (most ≥0.80), but the mere 2 studies with external exploratory (26, 30) showed a 15% average AUC reduction (0.677–0.81). Internal validation’s reliance on the same population leads to overfitting, and some studies highlighted optimal outcomes (XGBoost AUC = 0.919) while omitting less favorable ones (SVM AUC = 0.863). Unreported methodological details and absent calibration assessments further erode credibility, as illustrated by Liu et al. (30) XGBoost model (AUC = 0.941 in training vs. 0.724 in external exploratory).

To address these bottlenecks and transition AP-AKI prediction models from high AUC to high reliability, three key improvements are essential. First, ensure rigorous sample-predictor matching by adhering to the predictor number × 10 × 4 criterion: a 2-predictor model suits 110-participant studies, while 5 predictors require ≥200 cases to maintain adequate EPV. Second, standardize variable selection and modeling by abandoning univariate preselection in favor of literature-driven consensus + LASSO regression, prioritizing biologically plausible core predictors and complementing complex algorithms with multicenter external validation. Third, implement comprehensive methodological reporting aligned with relevant guidelines, disclosing blinding procedures, missing data handling, cross-validation parameters, and calibration metrics.

Current research on AP-AKI prediction models tends to exhibit a noticeable dichotomy: large-sample models generally demonstrate satisfactory robustness, while small-sample and complex models often face challenges related to EPV imbalance, variable selection bias, and selective reporting. To facilitate the translation of these models into clinical practice for early AKI warning in AP patients, future studies may benefit from prioritizing three key aspects: rigorous sample-predictor matching, standardized methodological reporting, and multicenter external validation. These measures could help more fully realize the clinical utility of AP-AKI prediction models, potentially bridging the gap between laboratory-derived high AUC values and real-world diagnostic reliability.

These methodological limitations must be prioritized when developing or refining predictive models to enhance their clinical value. Notably, none of the models included in this review have been widely implemented in clinical practice. The lack of multicenter data and external exploratory severely limits their generalizability and applicability.

Publication bias is a common limitation in systematic reviews and meta-analyses. Notably, the absence of gray literature may exacerbate such bias, as gray literature often contains preliminary findings, negative results, or small-sample-size studies—these studies are less likely to be published in peer-reviewed journals, which may lead to overestimation of the performance of predictive models for AKI in AP. To address this bias, our study strictly followed Cochrane recommendations: we systematically searched Chinese and English gray literature from multiple authoritative sources, and additionally contacted field experts to obtain unpublished data. Unfortunately, no eligible gray literature was included in the final analysis, primarily due to insufficient key information or inaccessible data; nevertheless, given current data accessibility, our comprehensive search minimized the risk of publication bias as much as possible.

Future studies should adhere to the PROBAST and TRIPOD guidelines (47) to ensure methodological rigor and transparent reporting, including documentation of missing data handling and variable selection rationale. Multicenter external validation across diverse healthcare settings is essential for enhancing model robustness and generalizability beyond single-center cohorts. Large-scale prospective studies are also needed to expand biologically plausible variables and optimize interpretability tools to improve clinician acceptance. For individual participant data (IPD)-based models, penalized regression methods should be adopted to mitigate overfitting and improve generalizability in scenarios with numerous variables or low EPV ratios. Notably, model calibration—often overlooked—must be systematically evaluated via metrics like Brier score or calibration plots, with recalibration strategies proposed for new populations.

Future research should clarify clinical decision thresholds and report DCA to quantify net clinical benefit, ensuring translation to improved patient outcomes rather than just statistical efficacy. Finally, model fairness and accessibility require subgroup analyses to avoid bias, with priority given to models relying on routinely available indicators for implementation in resource-limited settings.

This review is subject to several limitations that warrant consideration: First, our inclusion criteria were restricted to peer-reviewed studies published in Chinese and English, potentially introducing publication bias by overlooking relevant research in other languages and omitting unpublished studies with negative or neutral findings. Second, there is considerable heterogeneity among the included studies, stemming from variations in patient demographic profiles, modeling approaches, and operational definitions of AP-AKI; this heterogeneity, in turn, limits meta-analysis to a subset of models, hindering in-depth investigation of the factors driving heterogeneity and robust evaluation of publication bias through methods such as funnel plot interpretation and Egger’s test. Finally, while we systematically reviewed 37 predictive models for AP complicated by AKI, all included studies were assessed as carrying a high risk of bias via the PROBAST tool. Key sources of bias primarily involve insufficient reporting of methodological details, lack of systematic calibration assessment, and inconsistencies in outcome measure reporting—factors that collectively impose notable constraints on the clinical applicability and generalizability of these models.

## 5 Conclusion

Prediction models for AP complicated by AKI demonstrated moderate overall performance in terms of discrimination, calibration, and potential clinical utility. However, all included studies were assessed as having a high risk of bias, and many models lacked adequate internal validation and external validation. These limitations highlight the need for further refinement and rigorous evaluation before clinical implementation. Eleven predictors were consistently identified across models and should be prioritized in future predictive model development and validation.

## Data Availability

The original contributions presented in the study are included in the article/Supplementary material, further inquiries can be directed to the corresponding authors.

## Footnotes

1
https://www.math.hkbu.edu.hk/~tongt/papers/median2mean.html
