# Supplementary material for: Predictive models for acute kidney injury in acute pancreatitis: a systematic review and meta-analysis
Source: Front Med (Lausanne). 2026 Feb 5;12:1699717. doi: 10.3389/fmed.2025.1699717 (PMC12917899; doi:10.3389/fmed.2025.1699717)
Supplement: Supplementary file 1 [file Table_1.DOCX]

Supplementary Material

# Supplementary Material Table 1 Summary Table of Key Characteristics for Clinical Usability of AP-AKI Predictive Models

| **Included literature** | **Year of publication** | **Country** | **Study design** | **Sample size** | **AKI incidence (%)** | **Model input indicators & Accessibility¹** | **Measurement time point²** | **Decision threshold³** | **Net benefit analysis (DCA)⁴** |
| --- | --- | --- | --- | --- | --- | --- | --- | --- | --- |
| Yang et al. | 2022 | China | Retrospective study | 996 | 20.28 | IAP (Partially available), CRP (Routinely available), CysC (Partially available) | Within 24h after admission | Not reported | Conducted (Net benefit not specified) |
| Qu et al. | 2020 | China | Retrospective study | 334 | 23.95 | APACHE II (Routinely available), IAP (Partially available), PCT (Routinely available) | Within 48h after admission | Not reported | Conducted (Net benefit not specified) |
| Lin et al, | 2019 | China | Retrospective study | 308 | 26.95 | CRP (Routinely available), ALB (Routinely available), APACHE II (Routinely available), PO₂ (Routinely available), CA²⁺ (Routinely available) | Within 24h after admission | Not reported | Not conducted |
| Lu et al. | 2022 | China | Retrospective study | 295 | 20.68 | APACHE II (Routinely available), Ranson (Routinely available), Scr (Routinely available), PCT (Routinely available), CysC (Partially available), CA²⁺ (Routinely available) | Within 24h after admission | Not reported | Conducted (Significant net benefit in high-risk population) |
| Yang et al. | 2022 | China | Retrospective study | 424 | 15.80 | CRP (Routinely available), PLR (Routinely available), NAR (Routinely available), NLR (Routinely available), Scr (Routinely available), CysC (Partially available) | Within 24h after admission | Not reported | Not conducted |
| Jiang et al. | 2023 | Israel | Retrospective study | 963 | 72.17 | Weight (Routinely available), Sepsis (Routinely available), CHF (Routinely available), SOFA (Routinely available), Wbc (Routinely available), Alb (Routinely available) | Within 24h after admission | Not reported | Conducted (Brier score reported, net benefit not detailed) |
| Zhu et al. | 2023 | China | Retrospective study | 450 | 13.78 | Male (Routinely available), SAP (Routinely available), Hypoproteinemia (Routinely available), Diabetes (Routinely available), Obesity (Routinely available) | Within 24h after admission | Not reported | Not conducted |
| Li et al. | 2023 | China | Retrospective study | 437 | 32.04 | CRP (Routinely available), IAP (Partially available), CysC (Partially available) | Within 24h after admission | Not reported | Not conducted |
| Wu et al., | 2023 | Israel | Retrospective study | 799 | 62.45 | Age (Routinely available), Ethnicity (Routinely available), T-BIL (Routinely available), APTT (Routinely available), Vasoactive drugs (Routinely available), Sepsis (Routinely available) | Within 24h after admission | Not reported | Not conducted |
| Sun et al., | 2024 | China | Retrospective study | 249 | 25.70 | Scr (Routinely available), BUN (Routinely available), CRP (Routinely available), NLR (Routinely available), APACHE II (Routinely available) | Within 24h after admission | Not reported | Not conducted |
| Chi et al., | 2024 | China | Retrospective study | 258 | 30.62 | Age (Routinely available), TyG (Routinely available), PCT (Routinely available) | Within 24h after admission | Not reported | Not conducted |
| Zhang et al. | 2024 | China | Prospective study | 772 | 48.45 | Cr (Routinely available), ALB (Routinely available), LHD (Routinely available) | Within 48h after admission | Not reported | Conducted (Net benefit not specified) |
| Chi et al. | 2024 | China | Prospective study | 258 | 30.62 | Age (Routinely available), TyG (Routinely available), PCT (Routinely available) | Within 24h after admission | Not reported | Conducted (DCA results reported, net benefit not detailed) |
| Lin et al., | 2024 | America | Retrospective study | 1235 | 54.00 | Urine volume (Routinely available), Mechanical ventilation (Routinely available), Wbc (Routinely available), Vasoactive drugs (Routinely available), Mean heart rate (Routinely available), Mean respiratory rate (Routinely available), Maximum creatinine levels (Routinely available) | Within 24h after admission | Not reported | Conducted (Significant net benefit in clinical threshold range) |
| Liu et al. | 2024 | China | Retrospective study | 1089 | 13.77 | Age (Routinely available), Neutrophils (Routinely available), RDW (Routinely available), BUN (Routinely available), AlB (Routinely available), SBP (Routinely available), RRT (Routinely available), Vasopressor (Routinely available) | Within 24h after admission | Not reported | Not conducted |
| Yuan et al. | 2024 | China | Retrospective study | 672 | 20.53 | Radiomics features (Partially available) | Within 48h after admission | Not reported | Not conducted |
| He et al. | 2024 | China | Retrospective study | 110 | 20.90 | VExUS Score (Partially available), TyG Index (Routinely available) | Within 24h after admission | Not reported | Not conducted |

Table Notes

¹ Accessibility classification:

Routinely available: Detectable in both primary and tertiary hospitals (e.g., CRP, Scr, APACHE II score, basic demographic indicators);

Partially available: Only provided by tertiary/specialized hospitals (e.g., IAP, CysC, VExUS Score, radiomics features).

² "Not specified" indicates no clear detection time window reported in the original literature; "Within Xh after admission" is extracted from the original study’s methodology section.

³ "Not reported" means the original literature only focused on predictive efficacy (AUC) without specifying the clinical practical threshold (e.g., optimal prediction probability cutoff).

⁴ "Conducted" indicates the original study reported DCA analysis; supplementary details are key conclusions from the original literature; "Not conducted" means no DCA-related content was mentioned.

# Supplementary Material Table 2 Table of Detailed Search Strategies for Grey Literature Databases

| Database Category | Database Name | Search Mode | Search Fields | Search Query/Terms | Language Filter | Time Range | Filter Criteria |
| --- | --- | --- | --- | --- | --- | --- | --- |
| Chinese Grey Literature Databases | CNKI Doctoral and Master's Dissertation Database | Advanced Search | Title, Abstract, Keywords | ("acute pancreatitis" OR "acute necrotizing pancreatitis" OR "AP") AND ("acute kidney injury" OR "AKI" OR "acute renal failure" OR "acute renal insufficiency") AND ("predictive model" OR "risk prediction model" OR "predictive score" OR "risk score" OR "prognostic model") | Chinese | From database inception to Nov 8, 2025 | Document Type: Doctoral dissertation, Master's dissertation |
|  | China Conference Paper Full-text Database (CNKI Version) | Advanced Search | Title, Abstract, Keywords | Same as above | Chinese | From database inception to Nov 8, 2025 | Document Type: Conference paper |
|  | China Conference Paper Full-text Database (Wanfang Version) | Advanced Search → Select "Conference Papers" | Title, Abstract, Keywords | Same as above | Chinese | From database inception to Nov 8, 2025 | Document Type: Conference paper |
|  | China National Medical Research Registry (ChiCTR) | Basic Search | Study Title, Study Protocol, Keywords | ("acute pancreatitis" OR "AP") AND ("acute kidney injury" OR "AKI") AND ("predictive model" OR "risk score" OR "prognostic model") | Chinese | From database inception to Nov 8, 2025 | Study Type: Observational study, Diagnostic test study; Status: Completed, Recruiting, Enrolling by invitation (exclude "Withdrawn") |
|  | National Science and Technology Achievements Network | Advanced Search → Select "Achievements" | Achievement Name, Abstract, Key Technology Words | ("acute pancreatitis" OR "AP") AND ("acute kidney injury" OR "AKI") AND ("predictive model" OR "risk prediction model" OR "prognostic model") | Chinese | From database inception to Nov 8, 2025 | Technology Field: Medical and Health |
| English Grey Literature Databases | OpenGrey | Advanced Search | Any field | ("acute pancreatitis" OR "acute necrotizing pancreatitis" OR "AP") AND ("acute kidney injury" OR "AKI" OR "acute renal failure" OR "acute renal insufficiency") AND ("predictive model" OR "risk prediction model" OR "predictive score" OR "risk score" OR "prognostic model") | English | From database inception to Nov 8, 2025 | Document Type: Technical report, Research report, Working paper |
|  | ProQuest Dissertations & Theses Global | Advanced Search | Title, Abstract, Keywords | Same as above | English | From database inception to Nov 8, 2025 | Document Type: Dissertation, Thesis |
|  | WHO Global Index Medicus (GIM) | Advanced Search | Title, Abstract, Mesh Terms | ("acute pancreatitis"[Title/Abstract] OR "AP"[Title/Abstract]) AND ("acute kidney injury"[Title/Abstract] OR "AKI"[Title/Abstract]) AND ("predictive model"[Title/Abstract] OR "risk score"[Title/Abstract]) | English | From database inception to Nov 8, 2025 | Source Type: Grey literature (Reports, Guidelines, Working papers) |
|  | ClinicalTrials.gov | Advanced Search | Condition or disease, Intervention or treatment, Other terms | Condition: "acute pancreatitis" OR "AP"; Other terms: "acute kidney injury" OR "AKI" OR "predictive model" OR "risk prediction model" OR "prognostic model" | English | From database inception to Nov 8, 2025 | Study Type: Observational, Diagnostic; Study Status: Completed, Active (not recruiting), Recruiting; Result Filter: Include studies with/without results |
|  | EU Clinical Trials Register | Advanced Search | Disease/condition, Other terms | Disease/condition: "acute pancreatitis" OR "AP"; Other terms: "acute kidney injury" OR "AKI" OR "predictive model" OR "risk score" | English | From database inception to Nov 8, 2025 | Study Type: Observational study; Status: Completed, Ongoing |
|  | NIH Internal Research Reports (via NIH Public Access) | Basic Search | - | ("acute pancreatitis" OR "AP") AND ("acute kidney injury" OR "AKI") AND ("predictive model" OR "risk prediction model" OR "prognostic model") AND ("internal report" OR "research report" OR "unpublished") | English | From database inception to Nov 8, 2025 | Institution: National Institutes of Health (NIH) |

Note: AP = acute pancreatitis; AKI = acute kidney injury. "Same as above" refers to the search query used in "CNKI Doctoral and Master's Dissertation Database" (Chinese) and "OpenGrey" (English), respectively.

# Supplementary Material Table 3 Table of Search Strategies for Target Databases

| Database Category | Database Name | Abbreviation | Search Strategy |
| --- | --- | --- | --- |
| Chinese Databases | China National Knowledge Infrastructure | CNKI | SU=((Acute Pancreatitis OR Acute Necrotizing Pancreatitis OR AP) AND (Acute Kidney Injury OR AKI OR Acute Renal Failure OR Acute Renal Insufficiency) AND (Predictive Model OR Risk Prediction Model OR Predictive Score OR Risk Score OR Prognostic Model)) |
|  | Wanfang Data | Wanfang | (Title:Acute Pancreatitis OR Title:Acute Necrotizing Pancreatitis OR Title:AP) OR (Abstract:Acute Pancreatitis OR Abstract:Acute Necrotizing Pancreatitis OR Abstract:AP) OR (Keywords:Acute Pancreatitis OR Keywords:Acute Necrotizing Pancreatitis OR Keywords:AP) AND (Title:Acute Kidney Injury OR Title:AKI OR Title:Acute Renal Failure OR Abstract:Acute Kidney Injury OR Abstract:AKI OR Abstract:Acute Renal Failure OR Keywords:Acute Kidney Injury OR Keywords:AKI OR Keywords:Acute Renal Failure) AND (Title:Predictive Model OR Title:Risk Score OR Abstract:Predictive Model OR Abstract:Risk Score OR Keywords:Predictive Model OR Keywords:Risk Score OR Title:Prognostic Model OR Abstract:Prognostic Model OR Keywords:Prognostic Model) |
|  | VIP Database | VIP (Wipro) | (TI=Acute Pancreatitis OR TI=Acute Necrotizing Pancreatitis OR TI=AP OR AB=Acute Pancreatitis OR AB=Acute Necrotizing Pancreatitis OR AB=AP OR KY=Acute Pancreatitis OR KY=Acute Necrotizing Pancreatitis OR KY=AP) AND (TI=Acute Kidney Injury OR TI=AKI OR TI=Acute Renal Failure OR AB=Acute Kidney Injury OR AB=AKI OR AB=Acute Renal Failure OR KY=Acute Kidney Injury OR KY=AKI OR KY=Acute Renal Failure) AND (TI=Predictive Model OR TI=Risk Score OR AB=Predictive Model OR AB=Risk Score OR KY=Predictive Model OR KY=Risk Score OR TI=Prognostic Model OR AB=Prognostic Model OR KY=Prognostic Model) |
|  | Chinese Medical Association Database | CMA | (Subject Term:Acute Pancreatitis OR Subject Term:Acute Necrotizing Pancreatitis OR Free Term:AP) AND (Subject Term:Acute Kidney Injury OR Subject Term:Acute Renal Failure OR Free Term:AKI) AND (Free Term:Predictive Model OR Free Term:Risk Prediction Model OR Free Term:Risk Score OR Free Term:Prognostic Model) |
| English Databases | PubMed | PubMed | Basic Strategy: ("Acute Pancreatitis"[Title/Abstract] OR "Acute Necrotizing Pancreatitis"[Title/Abstract] OR "AP"[Title/Abstract]) AND ("Acute Kidney Injury"[Title/Abstract] OR "AKI"[Title/Abstract] OR "Acute Renal Failure"[Title/Abstract] OR "Acute Renal Insufficiency"[Title/Abstract]) AND ("Predictive Model"[Title/Abstract] OR "Risk Prediction Model"[Title/Abstract] OR "Predictive Score"[Title/Abstract] OR "Risk Score"[Title/Abstract] OR "Prognostic Model"[Title/Abstract])Optimized Strategy (with MeSH Terms): ((("Pancreatitis, Acute"[MeSH Terms] OR "Pancreatitis, Necrotizing"[MeSH Terms]) OR ("Acute Pancreatitis"[Title/Abstract] OR "AP"[Title/Abstract])) AND ((("Kidney Injury, Acute"[MeSH Terms] OR "Renal Insufficiency, Acute"[MeSH Terms]) OR ("Acute Kidney Injury"[Title/Abstract] OR "AKI"[Title/Abstract])) AND ("Predictive Models"[MeSH Terms] OR "Risk Assessment"[MeSH Terms] OR "Predictive Model"[Title/Abstract] OR "Risk Score"[Title/Abstract]))) |
|  | Web of Science | WoS | TS=("acute pancreatitis" OR "acute necrotizing pancreatitis" OR "AP") AND TS=("acute kidney injury" OR "AKI" OR "acute renal failure" OR "acute renal insufficiency") AND TS=("predictive model" OR "risk prediction model" OR "predictive score" OR "risk score" OR "prognostic model") |
|  | Scopus | Scopus | TITLE - ABS - KEY("acute pancreatitis" OR "acute necrotizing pancreatitis" OR "AP") AND TITLE - ABS - KEY("acute kidney injury" OR "AKI" OR "acute renal failure" OR "acute renal insufficiency") AND TITLE - ABS - KEY("predictive model" OR "risk prediction model" OR "predictive score" OR "risk score" OR "prognostic model") |
|  | Cochrane Library | Cochrane Library | #1: MeSH Terms "Pancreatitis, Acute" OR "Pancreatitis, Necrotizing" OR Text Words "acute pancreatitis" OR "acute necrotizing pancreatitis" OR "AP"#2: MeSH Terms "Kidney Injury, Acute" OR "Renal Insufficiency, Acute" OR Text Words "acute kidney injury" OR "AKI" OR "acute renal failure"#3: Text Words "predictive model" OR "risk prediction model" OR "risk score" OR "prognostic model"#4: #1 AND #2 AND #3 |
